# Acceptance patterns and decision-making for human papillomavirus vaccination among parents in Vietnam: an in-depth qualitative study post-vaccination

**DOI:** 10.1186/1471-2458-12-629

**Published:** 2012-08-09

**Authors:** Jane K Cover, Nguyen Quy Nghi, D Scott LaMontagne, Dang Thi Thanh Huyen, Nguyen Tran Hien, Le Thi Nga

**Affiliations:** 1 PATH, 2201 Westlake Avenue, Seattle, WA, USA; 2 PATH, Unit 01-02, Floor 2nd, Hanoi Towers, 49 Hai Ba Trung, Hoan Kiem District, Vietnam; 3 National Institute of Hygiene and Epidemiology, Ministry of Health, 1 Yersin St, Hanoi, Vietnam

**Keywords:** HPV vaccine, Adolescents, Acceptability, Decision-making

## Abstract

**Background:**

The GAVI Alliance’s decision in late 2011 to invite developing countries to apply for funding for human papillomavirus (HPV) vaccine introduction underscores the importance of understanding levels of HPV vaccine acceptance in developing country settings. In this paper, we present findings from qualitative research on parents’ rationales for vaccinating or not vaccinating their daughters (vaccine acceptance) and their decision-making process in the context of an HPV vaccination demonstration project in Vietnam (2008–2009).

**Methods:**

We designed a descriptive qualitative study of HPV vaccine acceptability among parents of girls eligible for vaccination in four districts of two provinces in Vietnam^a^. The study was implemented after each of two years of vaccinations was completed. In total, 133 parents participated in 16 focus group discussions and 27 semi-structured interviews.

**Results:**

Focus group discussions and in-depth interviews with parents of girls vaccinated revealed that they were generally very supportive of immunization for disease prevention and of vaccinating girls against HPV. The involvement of the National Expanded Program of Immunization in the demonstration project lent credibility to the HPV vaccine, contributing to high levels of acceptance. For parents who declined participation, concerns about side effects, the possibility that the vaccine was experimental, and the possible impact of the vaccine on future fertility rose to the surface. In terms of the decision-making process, many parents exhibited ‘active decision-making,’ reaching out to friends, family, and opinion leaders for guidance prior to making their decision.

**Conclusion:**

Vietnam’s HPV vaccination experience speaks to the importance of close collaboration with the government to make the most of high levels of trust, and to reduce suspicions about new vaccines that may arise in the context of vaccine introduction in developing country settings.

## Background

The burden of mortality from cervical cancer falls disproportionately upon women living in the developing world, where 88% of deaths from cervical cancer occur [1]. Cervical cancer is the second most common form of cancer among women in the developing world and the leading cause of cancer mortality for women. While cancer mortality has declined in high-income countries with improved screening and treatment services, the incidence of cervical cancer is projected to increase in middle- and low- income countries as their populations grow and age. By 2030, these countries will be home to an estimated 98% of cervical cancer deaths [1].

In light of the difficulty and cost of introducing effective and widespread screening services in low-resource settings, successful production of cervical cancer vaccines constitutes an important advancement for women's health. Currently, there are two vaccines—Merck’s Gardasil® and GlaxoSmithKline’s Cervarix®—that are highly effective in protecting against the strains of human papillomavirus (HPV) that cause 70% of cervical cancers. These vaccines have been licensed in more than 100 countries and, by the close of 2010, introduced in 33 national health programs. Though most of these national programs are in high-income countries, 20 developing countries have undertaken pilot projects to test the feasibility of introducing HPV vaccine on a national scale [2]. One such country, Vietnam, began testing strategies for HPV vaccine delivery in 2008, collecting data on coverage, acceptability, feasibility, and costs associated with integrating HPV vaccine into their National Expanded Program on Immunization (NEPI).

In low-resource settings, many factors influence the decision of whether and how to integrate a new vaccine into a national health program. Policymakers are increasingly asking for evidence of the impact a new vaccine is likely to have on communities as well as on the health care delivery system [3-5]. Based on research conducted in Latin America, Winkler et al. identified five distinct factors that shape the decision to introduce cervical cancer vaccines into public sector health programs: knowledge of cervical cancer incidence and its connection to HPV; affordability considerations; political will in the context of competing interests; the feasibility of vaccine delivery; and vaccine acceptability in the community [6]. It is this last decision-making factor—acceptability—that is the focus of this paper. As part of their decision-making process, policymakers want to know whether the vaccine will be accepted by those communities that will benefit, whether local opinion leaders will be supportive of a new vaccine, and whether support has been garnered among health providers who are asked to promote and deliver the new vaccine [7,8].

In a recent literature review of studies exploring factors affecting HPV vaccine acceptance among adult women considering vaccination for themselves, Black et al. observed that the extent of knowledge of HPV infection and its connection to cervical cancer, the perception of risk for HPV infection or cervical cancer, the cost of the vaccine, and misinformation about HPV vaccine were all factors associated with vaccine acceptability for adult women [9]. Although the individual studies differ in methodology and focus, the main conclusion was that women generally were receptive toward HPV vaccine. An important caveat is that this review focused on studies of HPV vaccine acceptance in industrialized settings.

Far fewer studies address acceptability in developing country settings, prompting a call for further research [10]. A systematic review of HPV acceptability in nine countries in Asia Pacific included studies from India, Malaysia, Vietnam, Taiwan, Thailand, and South Korea (as well as Australia, New Zealand, and China) [11]. Despite the wide variation in methodology and approach, acceptability factors that emerged across a multitude of studies include perceived susceptibility to HPV (eight studies), perceived safety and side effects of the vaccine (twelve studies), concerns over effects on fertility (three studies), efficacy of the vaccine (six studies), and HPV vaccine recommendation by physician or social referent (seven studies). Research from Uganda presented similar areas of concern from parents [12], suggesting that motivating factors for parents related to HPV vaccine acceptance may be more universal than previously thought.

However, previous studies, including the reviews cited above, focus on “intent to vaccinate” prior to HPV vaccine availability or introduction. In Vietnam, we had the opportunity to examine parents’ rationales for either having or not having their daughters vaccinated and their decision-making process in the context of an on-going HPV vaccination demonstration project. This project, implemented by the National Institute for Hygiene and Epidemiology (NIHE), was designed to identify appropriate strategies for HPV vaccine delivery that could be integrated into NEPI. It targeted girls in grade 6 or 11 years old in four districts of two provinces, spanning urban, rural, and mountainous areas. The districts were selected based on cervical cancer disease burden, population size, geography, socioeconomic status, EPI performance, and staff capacities.

Most research on vaccine acceptability utilized a single type of measurement in exploring vaccine acceptability, often either a yes/no question [13,14] or multiple items with a Likert scale [15-18]. Measures derived solely from quantitative survey data may not be sufficient for understanding the many facets of acceptability [19] and these types of measures are best employed in tandem with qualitative approaches. In this article, we present findings from qualitative research on parental reasons for HPV vaccine acceptance/non-acceptance and their decision-making process after both the first and second year of HPV vaccine implementation in Vietnam, and contextualize these qualitative data with recently published quantitative data on vaccine acceptability from this same project [20].

## Methods^d^

We designed a descriptive qualitative study of HPV vaccine acceptability among parents of girls eligible for vaccination in four districts of two provinces in Vietnam.^a^ Eligible girls for HPV vaccination were either 11 years of age (for the health-center based delivery strategy) or in grade 6 of primary school (for the school-based delivery strategy), of which more than 80% were 11 years of age, approximately 13% were 10 years old, and the remaining were 12 years old [20]. The study was implemented after the first year of vaccinations ended in June 2009 and repeated after the second year of vaccinations was completed in April 2010.

We used focus group discussions (FGD) and semi-structured interviews (SSI) to explore reasons for HPV vaccine acceptance or non-acceptance as well as to ascertain the process by which parents make their decision. FGDs were conducted with parents of fully vaccinated girls. Due to the very small number of girls who were partially vaccinated and the concentration of non-vaccinated girls in one urban location where school-based delivery was implemented,^b^ SSIs were conducted with parents of non- or partially-vaccinated girls. Additionally, in the Vietnamese context, not participating in a community-based vaccination program may have resulted in feelings of stigmatization among parents, inhibiting discussion of the reasons for their decision in a group setting. Individual interviews with these parents provide a more neutral, non-threatening environment to explore their decision. The key areas of inquiry included in the FGD guide and the SSI questionnaire were the same for both groups of parents. Key themes to elicit information about vaccine acceptability and the decision-making process were explored through a series of open-ended questions; some examples are provided below.

Questions for FGDs:

· What factors (personal, family, community, information, communication strategy) or individuals influenced your decision to have your daughter vaccinated?

· Please tell me how you made the decision about your daughter’s vaccination. Who raised the issue of HPV vaccination first, then who said what, and, who gave the final decision? Who, if anyone, did you discuss this with?

· Did anyone help you decide on whether to have your daughter vaccinated? Who influenced, why, how, and when?

Questions for SSIs:

· Why did your daughter not receive the full three doses?

· Were there any concerns that you had that kept you from taking your daughter (back) for vaccination?

· Please tell me how you made the decision about your daughter’s vaccination. Who raised the issue of HPV vaccination first, then who said what, and, who gave the final decision? Who, if anyone, did you discuss this with?

· Did anyone help you decide on whether to have your daughter vaccinated? Who influenced, why, how, and when?

### Sampling process

**Figure 1:**
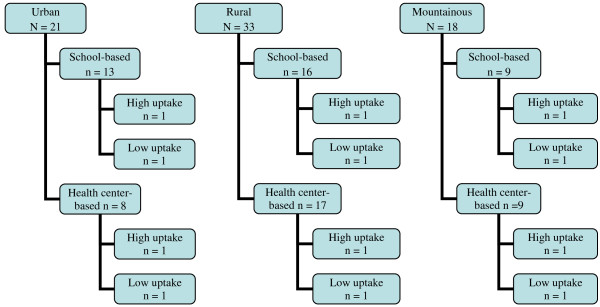
Sampling frame for acceptability by geographic area, strategy, and commune, Year 1.

The sampling process for study areas and populations varied from the first and second years of our study. As no study had yet been done of HPV vaccine acceptability after implementation of a HPV vaccination program, we selected a wide range of areas and large number of participants for our study after the first year of vaccinations. We performed criteria-based sampling for study areas using HPV vaccine delivery strategy implemented, type of geography (urban, rural, mountainous), and estimates of HPV vaccine uptake from NEPI reports. We used communes, administrative boundaries for health and other services in Vietnam, as the demarcation for study areas. All 72 communes that participated in the first year of the HPV vaccination program were classified by delivery strategy, type of geography, and low- or high-uptake based on NEPI reports (Figure 1). In each “geography-strategy” stratum, the three communes having the lowest uptake and the three with the highest were included in the sampling frame. From each group of three, one was randomly selected. The final sampling frame included 12 communes: ten communes had one FGD with parents of girls who received all three doses of HPV vaccine (fully vaccinated); and two communes with low uptake had SSIs with parents of girls who either were not vaccinated or received less than three doses (partially vaccinated). The rationale for sampling in this fashion was to ensure the inclusion of a range of different types of communes to reflect the geographic diversity of the country and the infrastructure capacities of communes that delivered HPV vaccines.

**Figure 2:**
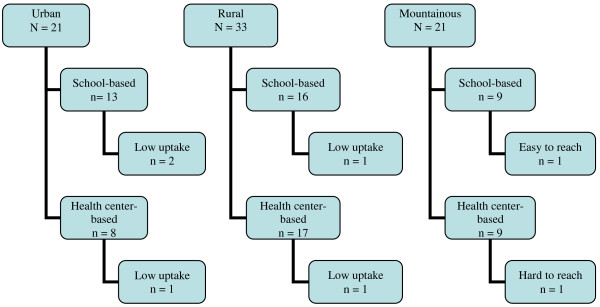
Sampling frame for acceptability by geographic area, strategy, and commune, Year 2.

The sampling process after the second year of vaccinations was different, as nearly all 72 communes had achieved high uptake during the second year of program implementation and there was a purposive attempt to oversample communes where vaccine uptake was the lowest. Again using communes as the area of study and stratifying by delivery strategy, in mountainous areas, we used geographic access as a selection criteria for identifying one ‘easy to reach’ commune (less than ten kilometers from district center) from the school-based delivery strategy and one ‘hard to reach’ commune (more than 40 kilometers from district center) from the health center-based delivery strategy out of a total of 18 communes. In rural areas, we randomly selected two communes (from a total of 33 communes), one from each delivery strategy, from a list of seven that did not report 100% HPV vaccination uptake at dose two. In urban areas, two communes (from a total of 21 communes), one from each delivery strategy, were purposively selected based on the highest reported number of girls not vaccinated at dose two (Figure 2). An additional commune with the lowest vaccine uptake during the first year of implementation from the school-based delivery strategy was added to the sampling frame for parents of partially/non-vaccinated girls to investigate changes in this area over time (not included in Figure 2). The final sampling frame included seven communes, six of which had one FGD each with parents of fully vaccinated girls and the seventh had parents of partially/non-vaccinated girls who were selected for a SSI. This sampling strategy allowed us to focus in on any regional differences, as well as to highlight reasons for non-acceptance of HPV vaccination.

### Participants and sample size

For all FGDs with parents of fully vaccinated girls, we used a purposive selection process to ensure a diversity of voices invited to participate in our study. Parents of fully vaccinated girls were selected by research staff from a list compiled by health workers, following the third dose of vaccination. Each FGD could accommodate ten to twelve participants. Similarly, participants in SSIs were selected from a list of partially/non-vaccinated girls.

After the research staff selected parents to invite to the FGD, they were contacted by commune health workers through household visits, given the invitation to participate, and informed of the date, time, and venue for the FGD. The same procedure was followed to invite parents of partially/non-vaccinated girls to a SSI. The final sample included 133 parents of girls eligible for HPV vaccination from 19 communes (Table 1), approximately two-thirds were mothers^c^.

**Table 1:** Study population, data collection method, and sample size for acceptability study, HPV vaccines, Vietnam, 2009–2010

	**Study population**	**Data collection method**	**Sample size**
Year 1	Parents of fully vaccinated girls	10 FGDs (10 communes)	64
	Parents of partially or non-vaccinated girls	24 SSIs (2 communes)	24
Year 2	Parents of fully vaccinated girls	6 FGDs (6 communes)	42
	Parents of partially or non-vaccinated girls	3 SSIs (1 commune)	3
Total		16 FGDs and 27 SSIs	133 parents

FGD focus group discussion; SSI semi-structured interview.

### Data collection, management, and analysis

Data were collected within two months of the last HPV vaccination session in each year of the program. All FGDs and SSIs were conducted in Vietnamese and tape recorded. Two members of the research team took part, with one serving as facilitator and the other as note taker. Notes taken during the discussion were used for reference and comparison during analysis. All tape recordings of FGDs and SSIs were transcribed. The unit of analysis was the parent.

Transcriptions were compared with notes taken during the discussion/interview as a preliminary data analysis stage to develop a standardized coding scheme [21,22]. NVivo electronic textual management software was used to organize, sort, and synthesize information into textual matrices based on the coding scheme developed for analysis and interpretation [23,24].

Both inductive and deductive analysis processes linked codes (representing key themes) to research questions. Multiple iterations of data synthesis, interpretation, and summarization were carried out. Using a reflective process, each iteration included a critical review of the data for emerging findings to further verify the data findings, followed by further refinement and synthesis of data [22]. This iterative process continued throughout the analysis.

Quality control procedures were implemented at several stages during data collection, management, and analysis. Research teams were comprised of two interviewers who compared notes and initial impressions after each FGD or SSI was completed. A standardized coding scheme of transcribed data ensured consistency in data organization and management, and facilitated a systematic approach to the data analysis to keep focused on the study objectives [22]. Validation of coding assignments on a random sample of FGDs by an independent qualitative researcher not directly involved in the data collection was also conducted to verify accuracy of coding by the research team.

### Ethics approvals

Verbal consent was a prerequisite for parents’ involvement, both for focus group participation and in the SSIs. A member of the research team served as a consent witness for parents. The research team explained to participants the purpose of the research, how they were invited, and the voluntary nature of their participation. No parent invited to participate refused. We do not have any reason to believe that our subjects were unusual in some way; however, purposive sampling techniques are not the same as random sampling and may not be representative of the entire population of parents whose daughters were eligible for HPV vaccine during the two-year program.

Our study was approved by the Research Ethics Committee at PATH (USA) and the Institutional Review Board at National Institute for Hygiene and Epidemiology (Vietnam).

## Results

### Reasons for vaccination

#### Perception of risks and health benefits

Parents reflected on the success of vaccinations in Vietnam in general, with a focus on disease prevention more broadly. As the following quote suggests, some parents participated in the HPV vaccination program less out of fear of HPV infection or cervical cancer per se than from a conviction that vaccination itself is beneficial:

“…Immunization is the best way of prevention. Whatever disease like tuberculosis or measles, immunization is the best. Prevention is better than treatment.”

Parent of fully vaccinated girl, FGD, Year 1, Rural, low uptake commune"

Others voiced health rationales that were specific to fears of cervical cancer:

"“I was advised by the doctor so I took the girl to get vaccination. It is because I saw many people had that awful disease. Most patients with cancer do not survive. That’s what I heard. I also knew that vaccination can prevent the disease ; that is why when hearing about vaccination, I was very happy.”

Parent of fully vaccinated girl, FGD, Year 2, Urban, low uptake commune

"Although health benefits were offered as a reason among participants across all regions, in focus groups in urban areas parents tended to specifically emphasize this point, confirming the importance of vaccination in preventing disease.

#### Trust in governmental program

Focus group findings also suggested that people were influenced by knowledge that the campaign was a publicly-implemented program, their reasoning grounded in a belief that “government cannot harm people’s health” with the introduction of a given health service or new vaccine.

"“Firstly, it is a government program. Secondly, it is the responsibility of all parents. Those reasons are enough to decide.”

Parent of fully vaccinated girl, FGD, Year 1, Rural, low uptake commune

This kind of statement was more common in the mountainous region, where many parents considered vaccine being delivered through the national health system as their primary reason for vaccination. They believed that they/their girls were selected to participate in the program because the government cared for them.

"“We heard people talking or saying on the loudspeaker that it was a dangerous disease which could lead to death if treatment did not work. Previously, we did not have opportunity to get treatment, so we could only foresee death. Now, the government cared for us, we shall take our daughter to get vaccinated.”

Parent of fully vaccinated girl, FGD, Year 2, Mountain, hard to reach commune

This may be due to the fact that people in mountainous areas, where most ethnic minorities live, receive several special supports from the government. When a government program is launched, they tend to participate in large numbers more so because the program is government sponsored.

#### Economic benefits

While the cost of HPV vaccination was not the most common motivation, it emerged as a theme in the FGDs with parents of fully vaccinated girls. Particularly among participants from rural areas, there was a tendency to focus on economic benefits (an expensive but freely-delivered vaccine). As the following quote illustrates, they emphasized that, in the context of high rural poverty, the free vaccine helped to increase acceptability.

"For those who are living in the rural area like us, we are still very poor. When we were told that vaccination helped women to prevent diseases, we found that it was really helpful. In fact, in the rural area, we cannot afford to take a child or a family member to the hospital for health care services. So when we heard that information on the loudspeaker and even on television, we found it did bring benefits and we took the girls to get vaccination.

Parent of fully vaccinated girl, FGD, Year 2, Rural, low uptake commune

However, in a larger context of vaccine acceptance, the economic rationale was not a critical factor.

### Reasons for non-acceptance of vaccination

Patterns for non acceptance of HPV vaccination reflected in qualitative research followed three principal themes: vaccine safety and side effects, suspicion and misconceptions about the HPV vaccine, and concerns related to the age of the girl and her risk of cervical cancer. The quote below, while touching on *vaccine safety and side effects*, also shows concern about the newness of the vaccine related to the daughter’s perceived vulnerable age:

"“Generally, I don’t need any more information. However, I worried if there would be any side effect on my daughter’s health [after vaccination]. I understood, but firstly, the vaccine is new. Secondly my daughter is still too young so I don’t want her to receive a new vaccine. Don’t know what will happen to her in the future.”

Parent of non vaccinated girl, SSI, Year 1, Urban, low uptake commune

This quote illustrates the interconnected nature of parent concerns and suggests that multiple issues may be raised when parents are given the opportunity to discuss their rationales in depth.

Another theme that emerged was *suspicion and misconceptions* about the program or vaccine, sometimes centered on mistrust that a very expensive vaccine was being offered free of charge and therefore may be low quality (“fake medicine”), and at other times expressed as fear that children may be part of a vaccine trial (primarily in the first year).

"“…What that professor said significantly influenced my decision. Working in health sector, he should be very knowledgeable in this issue. His remarks made me concerned, leading me to cancel the second dose[for my daughter]. People said this vaccine is very expensive, priority should go to relatives of ‘insiders’ who are highly ranked officers working in health sector. Secondly, if this vaccine is good, besides the option of having our daughter vaccinated for protection, we also have the choice of not taking it. In our situation, I felt some suspicion as my daughter’s teacher mentioned about various things [about the vaccine], scaring her…”

Parent of non vaccinated girl, SSI, Year 2, Urban, low uptake commune

"*"“On that day, there was an official from district and commune levels. I also asked questions, but they gave only general answers. They could not answer my question if this vaccine project is a trial or not, and who would take responsibility for the potential effects.”*

Parent of non vaccinated girl, SSI, Year 1, Rural, low uptake commune

"If the vaccine is introduced in the whole country then I have no objection, but why only two provinces, that is my concern. I feared that my daughter may be used for experiment; besides, I wonder why only children in grade 6 but not children of other ages? That did not convince me; maybe the vaccine is on trial and the children are used to check the effects of vaccine.”"

-Parent of non-vaccinated girl, SSI, Year 1, Urban, low uptake commune)

*Age-related concerns*—exposing young adolescent girls to HPV vaccination—were an important reason for refusal among some parents. From the quote below, it appears that not all parents understood that HPV vaccine has greater efficacy if administered prior to the onset of sexual activity. From the point of view of one mother, it was not necessary to administer HPV vaccine at such an early age, and better to wait until the girl reached maturity.

"“From what I have read, I found that girls at this age group have not started sexual activities, so the vaccine would only be effective in five years. After that, the girl should repeat doses each year. My girl is just 12 years old, which means that she will become sexually active in the next six years and will be exposed to the disease then. So I decided that whenever her health is ready, I would allow her to get vaccination. At that time, I believe that the vaccination program is better, so I would volunteer to take my daughter to preventive medical centers for vaccination.”

Parent of non-vaccinated girl, SSI, Year 2, Urban, low uptake commune

For other parents, concerns regarding the daughter’s age center on her perceived vulnerability, and a potential impact on her physical development.

"“In my girl’s case, she is not mature yet. She even has not started menstruation yet. I speak so truthfully to you because you are working in the health sector. You know, I am afraid whether vaccination would affect her natural development. I am afraid so I do not allow the girl to get vaccination.”"

Parent of non-vaccinated girl, SSI, Year 2, Urban, low uptake commune

### Active and passive decision-making

**Figure 3:**
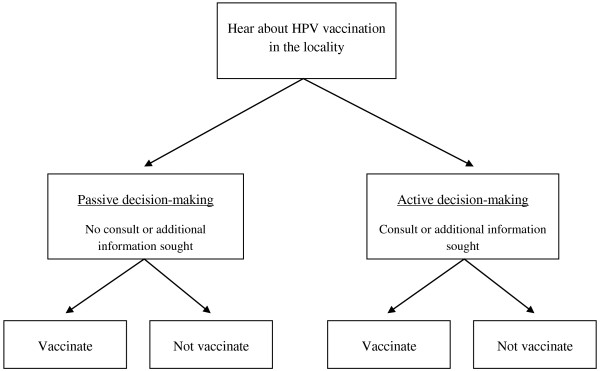
Decision- making process for HPV vaccine acceptance.

Based on analysis of how parents make decisions about HPV vaccination, we conceptualized the process as either a *passive decision* or *active decision* according to whether parents sought the advice of others. While the former refers to the group of parents who made the vaccination decision without seeking additional information, the latter includes those who decided after consulting one or more additional sources of information (see Figure 3).

#### Passive decision-making

Our qualitative findings suggest that passive decision-making occurred frequently during the first year of HPV vaccine implementation, and may have occurred more often in mountainous areas. The quote below illustrates this passive decision-making process:

I told my daughter that this was a deadly disease, and that my family was still in difficulty. As what was stated in the loudspeaker, vaccination was to prevent the girl from this disease when she grew up and got married. If the disease did occur, we could not afford the treatment. So it would be better if she got vaccination to prepare for perfect health in the future when she got married.

Parent of fully vaccinated girl, FGD, Year 2, Mountainous, hard to reach commune

Passive decision-making may prevail when parents participate in the HPV vaccination as a routine response; in the example below, vaccination is just something that one does as a matter of course:

"“…Immunization is the best way of prevention. Whatever disease like tuberculosis or measles, immunization is the best. Prevention is better than treatment.”

Parent of fully vaccinated girl, FGD, Year 1, Rural, low uptake commune

#### Active decision-making

On the contrary, people in urban areas tended to engage in more active decision-making, seeking additional information, often from individuals or sources outside the vaccination program, as illustrated below:

"“I searched in newspapers and found that although this vaccine was new in Vietnam, its use had been growing in many countries. So when I received the information, I talked to my wife and came to a final decision. I have two daughters, the older has mental problem, so I consider health as the most important issue for my younger girl. In South East Asia, cervical cancer is quite common, so when there is scientific advance in treatment, we, the parents, should create good conditions for our child. We both decided that.”

Parent of fully vaccinated girl, SSI, Year 1, Urban, low uptake commune

"“After the doctor said the vaccine has been circulated in the market for everyone already and passed the trial period, I felt more secure. Plus, my relatives said the vaccine has been used in America so that’s why I agreed to vaccinate my daughter.”

Parent of fully vaccinated girl, SSI, Year 1, Urban, low uptake commune

Media, with both positive and negative messaging, may be particularly influential for Active Decision-makers, as illustrated below:

"“I heard from people and from the radio that this vaccination was dangerous, and this and that bad things and this and that bad things may happen so I was afraid. But then my mother, mother-in-law, and cousins urged that I should take my daughter to get vaccinated, that we were still poor and it was a great and rare chance to get this donation, and that I should take the girl to get vaccinated for good health.”

Parent of fully vaccinated girl, FGD, Year 2, Urban, low uptake commune

"“In my case, when my daughter brought the vaccination invitation from school, I was also afraid. I felt afraid as some people who got rabies vaccine became paralytic after that. Those cases were also shown on television. I also did not know much about this vaccine, what kind of cancer it would prevent, so I was afraid that it might cause side effects like rabies vaccine. After careful consideration, I thought that for such expensive vaccine, which was sponsored and was developed by professional doctors and scientists would be not harmful. So I changed my mind and allowed the girl to get vaccinated. But at first I felt quite confused.”

Parent of fully vaccinated girl, FGD, Year 2, Urban, low uptake commune

The process of active decision-making takes time, as parents seek out additional resources and mull over their decision, as shown below. Parents with residual concerns may also wait to observe any negative consequences among vaccine recipients, before committing to vaccination:

"“…at the beginning I did not allow my daughter to receive vaccination. [However], after about a month, her school and the local health workers patiently invited me over to inform me about the HPV program. The doctors and nurses were very enthusiastic. If I had any questions or concerns or anything was unclear, they clarified these issues very thoroughly. Besides, I have relatives abroad who told me that where she lives now, they provide vaccination for women at the age of 25 instead of 11 years old like in Vietnam. Then, I was much more at ease and agreed to vaccinate my daughter. So, in fact, my daughter received the vaccination one month later than her friends.”

Parent of fully vaccinated girl, SSI, Year 1, Urban, low uptake commune

"“I find that those girls who were vaccinated last year, now they are in 7th grade and their health is still good. Last year, a counseling group visited each household to persuade the parents to have the girls vaccinated. Then the parents found that their girls were still in good health after vaccination. Thus this year, they even took the younger sister to get vaccinated.”

Parent of fully vaccinated girl, FGD, Year 2, Rural, low uptake commune

We found that parents rarely made decisions based on a single factor. As many of the quotes from our qualitative findings demonstrate, parents weighed a host of often interconnected considerations. They employed various strategies to reinforce their decision, including actively seeking further information, getting advice from health professionals or family members (in-country or abroad), or waiting and following other parents’ actions. Though some parents had unresolved concerns about side effects and safety, even after receiving information on HPV vaccination, most decided to accept vaccination based on advice and information from external sources.

## Discussion

In Vietnam, the EPI system has a long history of development and has met with considerable success in reducing the burden of vaccine-preventable diseases. Its strong reputation contributes to a positive attitude in the population toward vaccination in general. The Vietnam HPV vaccine experience suggests that NEPI has succeeded in building trust by demonstrating the effectiveness of vaccination for disease prevention. We found evidence of this in expressions of belief in vaccination in principle, and the identification of government involvement as a rationale for accepting vaccination. Indeed, formative research prior to the vaccine demonstration project found that Vietnamese parents, teachers, health providers, and civic leaders voiced high levels of confidence in the government immunization program [5,19]. For the general population in Vietnam, governmental programs are associated with high quality standards and rigor, so they are more likely to participate in these governmental programs rather than in seeking the same service from health providers in the private sector. The delivery of HPV vaccine by commune health center personnel and the additional involvement of local authorities brought credibility to the program and was a demonstration that the program was supported by the government.

In addition to finding common themes for vaccine acceptance between the first and second years of the program, our qualitative findings are also quite consistent with quantitative data from parental surveys conducted from the same vaccine demonstration project^d^. LaMontagne et al. found that Vietnamese parents were motivated to participate in the vaccination campaign by health rationales (92–94%), the involvement of the government (12–32%), the advice of health workers and teachers (3–36%), and to a lesser extent, by economic considerations (13–14%) (Table 2)^e^[20]. Health rationales were also a strong motivator for mothers participating in an HPV vaccine intent-to-vaccinate study recruited from a hospital in Da Nang [25]. However, this study was done prior to the availability of HPV vaccine in the Vietnamese market, discussed acceptance of HPV vaccine as an individual health care-seeking decision, and did not explore the principal role government plays as the provider of more than 90% of all vaccinations in the country.

**Table 2:** **Reasons for HPV vaccine acceptance, parents of fully vaccinated girls, 2009–2010 (Adapted**[20]**)**

**Rationales offered for acceptance**	**Year 1**		**Year 2**	
	**School (n=415)**	**Health center (n=263)**	**School (n=285)**	**Health center (n=219)**
Disease/infection prevention	52%	52%	76%	67%
Protection against cervical cancer	49%	44%	43%	51%
Vaccines are good for health	48%	42%	43%	44%
*Combined health related rationales*	*92%*	*92%*	*92%*	*94%*
Government program	20%	12%	29%	32%
Following advice of others	36%	3%	28%	19%
Vaccine is free	13%	14%	14%	14%
Knows someone who had cervical cancer	4%	4%	0%	0%

While health rationales were preeminent, 12% and 20% of survey respondents in year 1 and 29% and 32% in year 2 indicated that the program being run by the government was a motivating factor. This survey response pattern was particularly evident in the mountain and rural regions, where parents were significantly more likely to say they were influenced by the fact that it is a government program (p<.000; data not shown). These findings echo what we observed in the FGDs in the mountain region, where government involvement in the program was a strong motivating factor.

While the cost of HPV vaccination was not the most common motivation, it emerged as a theme in the FGDs with parents of fully-vaccinated girls, particularly among participants from rural areas. However, formative research in Vietnam found that even for non donated vaccine, acceptance is still high as long as the price is affordable [5]. Moreover, analysis of year 2 survey data by region found no geographic differences in the identification of cost factors as a rationale for vaccine acceptance, calling into question whether economic considerations are more prevalent in some parts of the country than others [20].

Rationales for not vaccinating that emerged in our qualitative study—fear of side effects, misconceptions and suspicions about HPV vaccine and the project, and age-related concerns—are also echoed in results from surveys of parents [20]. Rationales for not participating in the first year^f^ were concerns about the safety of HPV vaccine and possible side effects (33% and 6%), concerns about the health impacts of vaccination more generally (32% and 6%), and worries about the newness (15% and 12%) and possibly experimental nature of the vaccine (5% and 18%) (Table 3).

**Table 3:** **Reasons for not participating or not participating fully in HPV vaccination, 2009 (Adapted from**[20]**)**

**Rationales offered for partial or non vaccination**	**Year 1**	
	**School (n=85)**	**Health Center (n=17)**
Vaccine safety	33%	6%
Vaccination not good for health	32%	6%
Vaccine is new	15%	12%
Vaccine is experimental	5%	18%
Vaccine may impact fertility	12%	0%
School absenteeism (on vaccination day)	17%	12%
Not aware of program	6%	12%
Eligibility difficult to determine	2%	29%

Concerns about safety and vaccine side effects are a common refrain in HPV vaccine acceptability literature [11,26-28] but suspicion about the vaccine and the intentions of HPV program planners (reflected in parents’ concern that children may be part of a vaccine trial) emerged less often. In part, this reflects the preponderance of studies from industrialized settings with strong regulatory systems in place (e.g., US, UK, Australia, and Canada). Indeed, a number of studies from developing countries have reflected concern that HPV vaccines are not well-tested (South Korea), concern over whom the vaccine has been tested on (Ghana), and suspicion over the involvement of foreign entities (India) [29-31]. Collectively, these findings suggest that addressing suspicions related to foreign involvement and the difference between demonstration projects (or scale-up) and vaccine clinical trials may be crucial considerations for HPV program planning in developing country settings. The need to involve influential government stakeholders in a visible fashion is another lesson learned from PATH’s experience in Vietnam, and elsewhere [32].

While concerns about vaccine impacts on fertility were cited by less than 10% of parents of non-vaccinated girls, the findings from Vietnam echo those from other studies of HPV vaccine acceptability in developing country settings [33]. More than one third of Chinese parents (38%) cited age as a rationale for not vaccinating [34] and more than two thirds of Ghanaian respondents (68%) expressed concerns about HPV vaccine impacts on fertility [30]. Our qualitative findings suggest that worry over the vaccine undermining a girl’s ‘natural development’ and compromising her immature health status may be connected to concerns about compromising her future fertility. These findings suggest that formative research to explore cultural perceptions of adolescent health and development and how conceptions of adolescence as a vulnerable period may impact HPV vaccine uptake could be beneficial in advance of vaccine introduction. They also speak to the need for information, education, and communication efforts that target any perception that young female adolescents are especially vulnerable to negative health effects from vaccination, wherever such perceptions are identified.

With respect to the decision-making process, we identified many active decision-makers—parents who sought out additional information to inform and confirm their vaccination decisions. Indeed, findings from a quantitative study of vaccine acceptability showed that more than four out of five parents reported that they had discussed HPV vaccination with someone prior to their decision in the first year. As the vaccination campaign matured in the second year (and coverage rates reached 97%), there was still more communication and discussion about the program. In this study, 94% of surveyed respondents in Year 2 reported that they had discussed HPV vaccination with someone prior to making a decision [20]. Moreover, active decision-makers were significantly more likely to accept vaccination than passive decision-makers who made a decision without discussion (data not shown).

The high prevalence of active decision-making in Vietnam is quite consistent with results from the UK, where only 14% of parents based their HPV vaccine decision solely on information provided by the program [35]. However, that study identified a very intransigent population of active refusers, who, the authors felt, would be unlikely to change their minds despite exposure to additional information [35]. While these findings may suffer from selectivity bias, if active refusers are more likely to respond to the survey and attend information nights, the findings raise intriguing questions about the ability of vaccine campaigns to change entrenched anti-vaccine attitudes.

The existence of ‘active refuser’ populations who believe that vaccination may have negative, long-term, and unknown side effects is a widespread phenomenon. In the Netherlands, resistance to polio vaccination was sometimes based on religious conviction [36]. Highly educated parents may resist MMR vaccine on the belief that it ‘impairs the immune system’ [37]. Similar ‘anti-vaccination’ attitudes have been observed among parents with respect to HPV vaccine in Australia [38]. Indeed, anti-vaccination attitudes were common enough among parents in Australia that Cooper Robbins and colleagues, employing a conceptual framework similar in many respects to the model we use, identify ‘anti-vaccination’ as a separate decision-making state, distinct from active and passive decision-making. Our qualitative findings, in the context of a very high rate of vaccine uptake, particularly in the second year (97%) and among active decision-makers, suggest that such entrenched anti-vaccine (or anti-HPV vaccine) attitudes are yet to take root in Vietnam.

Our findings about the importance of active decision-making in a developing country setting generate several implications. First, to maximize effectiveness, communication campaigns may need to employ a broad brushstroke, targeting influential figures, teachers, health workers, and other members of the public; regardless of any direct involvement in the program, these individuals may influence uptake indirectly, through informal consultations. The second concerns the possibility that discussion with others may quell vaccine-related concerns and/or generate the perception of broader community support for vaccination. Researchers may want to explore further if and how dialogue facilitates decision-making and contributes to vaccine uptake. Third, program planners would do well to anticipate the need for active decision-making in the scheduling of vaccination campaign activities by providing information well in advance of the scheduled vaccination drive.

### Limitations and strengths

Our study, while robust, may have limitations that affect the interpretation of our findings. Even though we have tried for a diverse set of participants, because selection was criteria-based and not random, there could be selection bias among the participants that may over or under-estimate the different factors mentioned as motivators for vaccine acceptance.

We did not sample specifically for equal representation of mothers and fathers, and the sex of the parent was not available for parents of partially- or non-vaccinated girls who participated in a semi-structured interview. Therefore, we could not analyze our results for possible differences between parents. However, previous research about parental decision-making for vaccination in Vietnam indicated that such decisions are made jointly, suggesting that there would be a high concordance between parents’ acceptability, thus reducing the need to study mothers and fathers separately [5]. Previous HPV vaccine intent-to-vaccinate research among Vietnamese parents by Breitkopf, et al. demonstrated that mothers and fathers largely agreed on their recommendation to accept or not accept a hypothetical offer of HPV vaccine for their daughter [39].

Additionally, because so few girls overall were partially-vaccinated and only 3% of the total population did not accept HPV vaccine in year 2, there were too few parents available from these groups to constitute a focus group, necessitating the use of SSIs. While the SSI approach differs from a focus group, the SSI questionnaire content was nearly identical to that explored through the FGD methodology with parents of fully vaccinated girls that there may not have been a loss in substantive content through the two techniques. Even though our analysis utilized a rigorous inductive and iterative process to identify key themes and subthemes, we may have missed key minor voices, especially from parents of partially vaccinated girls, which could have enriched the picture of vaccine acceptability among parents in Vietnam. However, we sought to minimize these limitations by the extensive scope of our sample and thoroughness of our FGD and interview guides with parents, allowing for the broadest range of ideas to come forward during data collection. Additionally, the qualitative research methodology we employed allowed us to explore in greater depth parents’ stated reasons for participating or not participating in the vaccination campaign, to gain a more comprehensive understanding of the decision-making process itself—an in-depth exploration that is not necessarily possible through fixed-response survey questions utilized by quantitative techniques. Our qualitative methods complemented the quantitative surveys conducted among these same populations and they both reinforce/affirm/confirm each other, reflected in the consistency of results from both studies. Lastly, our study was able to confirm acceptability motivators and process by collecting data from parents at two points in time, after each year of vaccinations, which provided information on how HPV vaccine acceptability may have evolved over time as the vaccination program matured.

## Conclusion

Parents in Vietnam were motivated to allow their daughters to receive HPV vaccine by health rationales and by the involvement of the government (NEPI) in the vaccine program. Those who declined participation were influenced by concerns for vaccine safety and side effects, suspicions related to the newness and possible experimental nature of the vaccine, and concerns about negative health impacts of vaccinating adolescent girls. Like parents in high-resource settings, Vietnamese parents are active decision-makers, frequently seeking out additional sources of information to inform and confirm their vaccination decisions. Vietnam’s HPV vaccination experience speaks to the importance of close collaboration with the government to make the most of existing high levels of trust, and to reduce suspicions about new vaccines that are a natural component of vaccine demonstration projects. Cervical cancer continues to be a leading cause of death for women throughout the developing world. Experiences in Vietnam should be shared in a wider context to reduce the lag time between when a new vaccine becomes available in the market and when it is incorporated into national health programs in low-resource countries.

## Endnotes

^a^A separate study assessed vaccine acceptability among girls who were eligible for HPV vaccine. Results are presented in the following document: PATH and Vietnam National Institute of Hygiene and Epidemiology. *Evaluating HPV Vaccine Delivery Strategies in Vietnam*. Seattle, Washington: PATH; 2010.

^b^Of the 508 total eligible girls either not vaccinated or partially vaccinated, only 27 (5.3%) were partially vaccinated, which represents 0.8% of all girls who received the first dose of vaccine. Of the 481 total eligible girls not vaccinated in the first year, 73% resided in one urban area that implemented school-based delivery.

^c^The sex of the parent was collected only for FGD participants, so precise distribution of mothers and fathers is not available.

^d^We occasionally cite previously published findings from a representative survey of parents whose daughters were eligible for HPV vaccine during a demonstration project in Vietnam. A full description of this survey’s methodology has been previously published (LaMontagne DS *et al*., 2011).

^e^The data in Table 2 represents the range across the two delivery strategies (school-based and health center-based) and program years.

^f^Note that we replicate here only data published on the first year of the project, given very high coverage rates and only 14 surveyed parents with a partially- or non-vaccinated child in the second year.

## Competing interests

The authors declare no competing interests.

## Authors’ contributions

JKC synthesized collected data, contributed to data interpretation, substantively revised initial drafts of the manuscript, and led on the final revised manuscript. NQN was responsible for the study design, field data collection, data analysis, and authored the initial draft of the manuscript. DSL was the overall research manager of HPV vaccine acceptability studies across four countries, including Vietnam, contributed to the study design, critically reviewed data analysis and interpretation, and made substantive contributions to drafts of the manuscript. DTTH was responsible for the vaccine implementation in the study districts in Vietnam, provided input into the study design, reviewed preliminary results, and commented on the manuscript. NTH was the senior person in-charge of the vaccine implementation program in Vietnam, provided strategic leadership, reviewed and contributed to the study design, and commented on drafts of the results and manuscript. LTN was the principal investigator for the acceptability study, acted as the senior researcher in-charge in Vietnam, contributed to the study design, field data collection, data analysis, and made substantive contributions to drafts of the manuscript. All authors approved the final manuscript for submission.

## Pre-publication history

The pre-publication history for this paper can be accessed here:

http://www.biomedcentral.com/1471-2458/12/629/prepub
